# Synthetic mimetics assigned a major role to IFNAR2 in type I interferon signaling

**DOI:** 10.3389/fmicb.2022.947169

**Published:** 2022-09-02

**Authors:** Nele Zoellner, Noémi Coesfeld, Frederik Henry De Vos, Jennifer Denter, Haifeng C. Xu, Elena Zimmer, Birgit Knebel, Hadi Al-Hasani, Sofie Mossner, Philipp A. Lang, Doreen M. Floss, Jürgen Scheller

**Affiliations:** ^1^Medical Faculty, Institute of Biochemistry and Molecular Biology II, Heinrich-Heine-University, Düsseldorf, Germany; ^2^Medical Faculty, Institute of Molecular Medicine II, Heinrich-Heine-University, Düsseldorf, Germany; ^3^Medical Faculty, Institute for Clinical Biochemistry and Pathobiochemistry, German Diabetes Center, Heinrich-Heine-University, Düsseldorf, Germany

**Keywords:** interferon, synthetic, cytokine, virus, nanobody

## Abstract

Type I interferons (IFNs) are potent inhibitors of viral replication. Here, we reformatted the natural murine and human type I interferon-α/β receptors IFNAR1 and IFNAR2 into fully synthetic biological switches. The transmembrane and intracellular domains of natural IFNAR1 and IFNAR2 were conserved, whereas the extracellular domains were exchanged by nanobodies directed against the fluorescent proteins Green fluorescent protein (GFP) and mCherry. Using this approach, multimeric single-binding GFP-mCherry ligands induced synthetic IFNAR1/IFNAR2 receptor complexes and initiated STAT1/2 mediated signal transduction *via* Jak1 and Tyk2. Homodimeric GFP and mCherry ligands showed that IFNAR2 but not IFNAR1 homodimers were sufficient to induce STAT1/2 signaling. Transcriptome analysis revealed that synthetic murine type I IFN signaling was highly comparable to IFNα4 signaling. Moreover, replication of vesicular stomatitis virus (VSV) in a cell culture-based viral infection model using MC57 cells was significantly inhibited after stimulation with synthetic ligands. Using intracellular deletion variants and point mutations, Y510 and Y335 in murine IFNAR2 were verified as unique phosphorylation sites for STAT1/2 activation, whereas the other tyrosine residues in IFNAR1 and IFNAR2 were not involved in STAT1/2 phosphorylation. Comparative analysis of synthetic human IFNARs supports this finding. In summary, our data showed that synthetic type I IFN signal transduction is originating from IFNAR2 rather than IFNAR1.

## Introduction

Signal transduction of cytokines is executed by cytokine receptors which are in an off-mode without cytokine and a dimeric or multimeric on-mode after cytokine binding (Croxford et al., 2012). The on-state eventually became interrupted by depletion of the cytokine and cytokine receptor, by natural cytokine antagonists or intracellular negative feedback mechanisms. Monoclonal antibodies as synthetic cytokine antagonists are still a relatively recent but extremely successful development representing an own class of therapeutic biomolecules. Synthetic cytokine receptors are emerging tools for immunotherapeutic applications (Scheller et al., 2019), with chimeric antigen receptor (CAR) T-cell therapy being the first example which has been approved as gene therapy for the treatment of severe cases of acute lymphatic leukemia (Si et al., 2018).

Recently, we have developed a fully synthetic cytokine/cytokine receptor system which phenocopied cytokine signaling, exemplified for the pro-inflammatory cytokines Interleukin (IL-)6, IL-23, TNFα, the anti-inflammatory cytokine IL-22 and death ligand Fas (Engelowski et al., 2018; Mossner et al., 2020, 2021). This fully synthetic cytokine receptor system (SyCyR) is based on nanobodies specifically recognizing GFP and mCherry (Rothbauer et al., 2008; Fridy et al., 2014) fused to the transmembrane and intracellular domains of the receptor of interest. The nanobodies serve as extracellular sensors for homo- and heteromeric GFP-mCherry fusion proteins which induce receptor dimerization (Mossner et al., 2020). A nanobody or VHH domain consists of the N-terminal variable domain of Camelidae heavy chain antibody which is sufficient for antigen binding (Wesolowski et al., 2009). The major advantage of GFP and mCherry was that they have no human equivalent, enabling background-free and cell-type specific activation of synthetic cytokine receptors by dimeric GFP/mCherry ligands, e.g., of CAR T-cells functionally modified by SyCyRs. For human therapeutic adoptive transfer approaches of T-cells transduced with synthetic receptors, potential immunogenicity of GFP and mCherry may be a barrier because of the development of antibodies directed against these synthetic ligands. In addition, the SyCyR contains a non-natural extracellular component, which is the ligand-binding nanobody. Nanobodies can be humanized to reduce immunogenicity (Rossotti et al., 2022). Moreover, also the chimeric antigen receptor (CAR) is a fusion protein consisting of an extracellular non-natural ligand-binding single-chain Fv fragment and did not result in major immune rejection. Therefore, SyCyRs might be relatively safe.

Type I interferons (IFNs) are potent inhibitors of viral replication. The ability of type I IFNs to inhibit virus replication was first reported more than 60 years ago (Isaacs et al., 1957; Isaacs and Lindenmann, 1957). Currently, type I IFNs constitute one of the most powerful innate immune defense mechanisms known. Lack of type I IFN signaling results in excessive viral replication resulting in fatal viral infection (Muller et al., 1994). Consistently, type I IFN malfunctions are associated with life-threatening coronavirus disease 2019 (COVID-19) after SARS-CoV-2 infection (Zhang et al., 2020). Furthermore, patients exhibiting changes in type I IFN signaling can develop severe diseases after vaccination with live attenuated viruses causing such as measles, mumps, rubella (MMR) or yellow fever (Duncan et al., 2015; Hernandez et al., 2019). Because of their potent antiviral activity and their immune-stimulatory capacity, type I IFNs have been used clinically for the treatment of hepatitis C virus (HCV), and with limitation also for HBV (Degertekin and Lok, 2009). Apart from limiting viral replication, type I IFN can modify other immune cell subsets such as protection of cytotoxic T lymphocytes (CTLs) from natural killer (NK) cell–mediated regulation (Xu et al., 2014, 2017). However, a prolonged IFN signaling can be observed during chronic LCMV viral infection, this leads to up-regulation of PD-L1 and IL-10 on dendritic cells and macrophages, thereby contributing to the T-cell exhaustion (Teijaro et al., 2013; Wilson et al., 2013). Moreover, in HIV-1 infection, a decrease in CD4-T-cell is associated with an increase in IFN-I (type I IFN) level (Hardy et al., 2013; Dagenais-Lussier et al., 2017). Therefore, depending on the context, IFN-I can be beneficial or harmful to the host.

The type I IFN family consists of 13 IFNα subtype genes in humans (14 in mice), single IFNβ, IFNε, IFNκ, IFNω (humans), and IFNζ (mice) subtypes (Lazear et al., 2019; Mesev et al., 2019). Type I IFNs are known to bind to heterodimers of type I IFN receptors (IFNAR1 and IFNAR2) which mainly signal *via* the Janus kinase (Jak)/signal transducer and activator of transcription (STAT) signaling pathway. IFNAR1 and IFNAR2 are constitutively associated with tyrosine kinase 2 (Tyk2; Uzé et al., 1990; Colamonici et al., 1994) and Jak1 (Novick et al., 1994; Lutfalla et al., 1995), respectively, at well described interaction sites (Yan et al., 1996; Wallweber et al., 2014). The canonical type I IFN signaling pathway relies on the phosphorylation and nuclear translocation of STAT proteins, mainly STAT1 and STAT2, which became phosphorylated following receptor dimerization/activation. Together with IFN regulatory factor 9 (IRF9) they form the heterotrimeric complex IFN-stimulated gene factor 3 (ISGF3) to regulate the transcription of genes under control of IFN-stimulated regulatory elements (Platanias, 2005). STAT2 is constitutively associated with the cytosolic domain of IFNAR2 (Saleh et al., 2002) and STAT1 docking to IFNAR2 occurs through STAT1-STAT2 heterodimerization in agreement with the requirement of STAT2 for STAT1 phosphorylation by IFN (Li et al., 1997; Nguyen et al., 2002). The STAT2 interaction site is located within the last 110 amino acids of IFNAR2 (Shemesh et al., 2021). A very recent report illustrates the importance of the tyrosine residues in human IFNAR2 for STAT phosphorylation in general (Shemesh et al., 2021), albeit previous reports have also assigned these roles for tyrosine residues in IFNAR1 (Li et al., 1997). Of note, the activation of Jak–STAT pathway alone is not sufficient for the generation of complete biological activities of type I IFNs.

Here, we adopted our synthetic cytokine receptor technology to type I IFN signaling and showed synthetic IFN signaling and viral defense. Furthermore, mutation of critical tyrosine residues in synthetic IFNAR2 abolish STAT1/2 phosphorylation. Albeit with slightly lower efficiency, synthetic IFNAR2 homodimers also led to STAT1/2 phosphorylation.

## Materials and methods

### Cloning

The genes for type I IFN receptor chains were translated in one open reading frame using expression cassettes containing the foot-and-mouth disease virus (FMDV) 2A (F2A) self-processing sequence and synthesized by BioCat GmbH (Heidelberg, Germany). The cDNA was generated by fusion of coding sequences for human IL-11R signal peptide (SP; Q14626, aa 1–24) followed by sequences for HA tag (YPYDVPDYA), murine IFNAR2 (O35664, aa 22–513), furin cleavage site (RAKR) followed by sequence for F2A peptide (Fang et al., 2005), coding sequences for human IL-11R SP, myc tag (EQKLISEEDL) and murine IFNAR1 (P33896, aa 27–590). For synthetic myc-tagged GFP-nanobody-mIFNAR1 (V_G_mIFNAR1) or GFP-nanobody-hIFNAR1 (V_G_hIFNAR1) coding sequences of GFP-VHH (Rothbauer et al., 2008) and murine (S415 to C590) or human IFNAR1 (P17181, S422 to V557) have been fused representing 15 aa of the extracellular domain, the transmembrane domain and the cytoplasmic part of the receptor. For synthetic HA-tagged mCherry-nanobody-mIFNAR2 (V_C_mIFNAR2) or mCherry-nanobody-hIFNAR2 (V_C_hIFNAR2) coding sequences of mCherry-VHH (Fridy et al., 2014) and murine (I228 to R513) or human IFNAR2 (P48551, L229 to R515) have been fused representing 15 aa of the extracellular domain, the transmembrane domain and the cytoplasmic part of the receptor. The pcDNA3.1 expression vectors containing the cDNAs for the synthetic receptors were used as template for the generation of receptor deletion variants by standard PCR. Mutation of tyrosine to phenylalanine was generated by PCR using Phusion high-fidelity DNA polymerase, followed by DpnI digestion of methylated template DNA (Edelheit et al., 2009). For retroviral transduction of Ba/F3-gp130 cells expression cassettes were transferred into the retroviral vector pMOWS-puro (Ketteler et al., 2002). All generated expression plasmids have been verified by sequencing.

### Cells and reagents

The generation of Ba/F3-gp130 cells was described elsewhere (Chalaris et al., 2007). The packaging cell line Phoenix-Eco was received from Ursula Klingmüller (DKFZ, Heidelberg, Germany). HEK293 (ACC 305) cells were purchased from the Leibnitz Institute DSMZ-German Collection of Microorganisms and Cell Culture (Braunschweig, Germany). Kinase-deficient human fibrosarcoma cell lines U4C, γ2A, and U1A have been described (Behrmann et al., 2004). MC57 (CRL-2295) cells were from ATCC (Manassas, VA, United States). Cell lines were grown in DMEM high glucose culture medium (GIBCO^®^, Life Technologies, Darmstadt, Germany) supplemented with 10% fetal bovine serum (GIBCO^®^, Life Technologies), 60 mg/l penicillin and 100 mg/l streptomycin (Genaxxon bioscience GmbH, Ulm, Germany) at 37°C with 5% CO_2_. Proliferation of Ba/F3-gp130 cells was maintained in the presence of 0.2% (10 ng/ml) human Hyper-IL-6 (HIL-6; Fischer et al., 1997). Synthetic cytokine ligands were expressed and purified as described (Mossner et al., 2020). Recombinant murine IFNα4 (#12115–1) was obtained from pbl assay science (Piscataway, NJ, United States). Recombinant murine IFNβ [#8234-MB-010/*CF*), IFNγ (#485-MI-100) and IFNλ2 (#4635-ML-025) were purchased from R&D Systems (bio-techne, Minneapolis, MN, United States). Phospho-STAT1 [(Tyr701; 58D6), #9167], STAT1 (#9172), phospho-STAT2 [(Tyr690; D3P2P), #88410], STAT2 [(D9J7L), #72604], HA-Tag [(C29F4), #3724], Myc-Tag [(71D10), #2278], Jak1 [(6G4), #3344], Jak2 [(D2E12), #3230], Tyk2 (#9312) antibodies were obtained from Cell Signaling Technology (Frankfurt, Germany). Goat anti-rabbit IgG (H + L) cross-adsorbed secondary antibody, peroxidase-conjugated (#31462) was obtained from Pierce (Thermo Fisher Scientific, Waltham, MA, United States). Alexa Fluor 488 conjugated anti-rabbit IgG (H + L), F(ab’)_2_ fragment (#4412) was obtained from Cell Signaling Technology.

### Transfection of cells

HEK293, U4C, γ2A, and U1A cells (2 × 10^6^) were transiently transfected as indicated using TurboFect transfection reagent (Fermentas, Thermo Scientific) according to the manufacturer’s instructions. MC57 and Ba/F3-gp130 cells were retrovirally transduced with the pMOWS-puro expression plasmids as described (Floss et al., 2013). Transduced Ba/F3 cells were grown in DMEM medium as described above supplemented with 10 ng/ml HIL-6. Selection of transduced Ba/F3-gp130 and MC57 cells was performed with puromycin (1.5, 3 μg/ml; Carl Roth, Karlsruhe, Germany) for at least 2 weeks. Afterward, the generated MC57 and Ba/F3-gp130 cell lines were analyzed for receptor cell surface expression *via* flow cytometry.

### Cell surface detection of synthetic IFNARs *via* flow cytometry

Cell surface expression of stably transfected Ba/F3-gp130 and MC57 cells was detected by specific antibodies against HA and myc tag. 5 × 10^5^ cells were washed in FACS buffer (PBS, 1% BSA) and then incubated in 50 μl of FACS buffer containing the indicated specific primary antibody (Myc-Tag 1:100, HA-Tag 1:100). After incubation of at least 1 h at room temperature, cells were washed and resuspended in 50 μl of FACS buffer containing secondary antibody (Alexa Fluor 488 conjugated anti-rabbit IgG (H + L), F(ab’)_2_ fragment 1:100) and incubated for 1 h at room temperature. Cells were washed and resuspended in 500 μl of FACS buffer and analyzed by flow cytometry (BD FACSCanto II flow cytometer using the FACSDiva software, BD Biosciences). Data analysis was conducted using FCS Express 7 (De Novo Software, Pasadena, CA, United States).

### Cell viability assay

Ba/F3-gp130 cells were washed and 5 × 10^3^ cells were cultured for 3 days in a final volume of 100 μl in the presence or absence of cytokine/synthetic cytokine ligands. The CellTiter-Blue^®^ Reagent was used to determine cellular viability by recording the fluorescence (excitation 560 nm, emission 590 nm) using an Infinite M200 PRO plate reader (Tecan, Crailsheim, Germany) immediately after adding 20 μl of reagent per well (time point 0) and up to 120 min thereafter. All conditions were measured in triplicate per experiment. Fluorescence values were normalized by subtraction of time point 0 values. Data are presented as means ± SD. All experiments were performed at least three times, and one representative experiment was selected.

### Stimulation assays

Ba/F3-gp130 cells were washed and starved in serum-free medium for 4 h. Subsequently, cells were stimulated with the indicated ligands for the indicated time points, harvested by centrifugation at 4°C for 5 min at 1,500 rpm and frozen. Cells were lysed for 2 h in 10 mM Tris–HCl, pH 7.8, 150 mM NaCl, 0.5 mM EDTA, 0.5% NP-40, 1 mM sodium vanadate, and 10 mM MgCl_2_ supplemented with complete protease inhibitor cocktail tablets (Roche Diagnostics). Transiently transfected HEK293, U4C, γ2A, and U1A cells were washed and starved in serum-free medium overnight. MC57 cells were seeded 2 days prior to stimulation at density of 3-4 × 10^6^ cells per 10 cm cell culture dish. The next day, MC57 cells were washed with PBS and starved in serum-free medium overnight. Cells were stimulated with the indicated ligands for the indicated time points, harvested and lysed for 2 h in lysis buffer described above. Protein concentration of cell lysates was determined by the BCA Protein Assay (Pierce, Thermo Scientific). Analysis of STAT1/2 activation was performed by Western blotting of 50 μg of total soluble protein from total cell lysates and subsequent detection steps using the anti-pSTAT1/2 (1:1000) and anti-STAT1/2 (1:1000) antibodies described above.

### Western blotting

Proteins were separated by SDS-PAGE and transferred to polyvinylidene difluoride (PVDF) membranes. The membranes were blocked in 5% fat-free dried skimmed milk in TBS-T (10 mM Tris HCl pH 7.6, 150 mM NaCl, 1% Tween 20) and probed with the indicated primary antibodies in 5% BSA in TBS-T at 4°C overnight. After washing, membranes were incubated with secondary peroxidase-conjugated antibodies (1:2,500) diluted in 5% fat-free dried skimmed milk. The Immobilon™ Western Reagents (Millipore Corporation, Billerica, MA, United States) and the ChemCam Imager (INTAS Science Imaging Instruments GmbH, Göttingen, Germany) were used for signal detection.

### *In vitro* VSV replication assay

Equal amount of MC57-GFP and MC57-V_G_mIFNAR1-V_C_mIFNAR2 cells were seeded into 24 wells. The next day cells were treated with 100 ng/ml GC, 150 U/ml IFNα4 or left untreated, followed after 120 min by infection of VSV-Indiana at MOI 0.01 and 0.1. At 12 h or 24 h post-infection, virus in supernatant was analyzed for infectious virus using focus-forming assay on Vero cells (Lang et al., 2009).

### Microarray analysis

Ba/F3-gp130-V_G_mIFNAR1-V_C_mIFNAR2 cells were cultured as described before. Cells were washed four times with PBS to remove cytokines/synthetic cytokine ligands from the medium and then starved for 5 h in serum-free DMEM. Cells were stimulated for 240 min without cytokine or with 100 ng/ml GCCG or 100 U/ml IFNα4. Total RNA extraction of four independent biological replicates was carried out by an RNeasy Mini Kit (Qiagen, Hilden, Germany) according to manufacturer’s instructions. The microarray analysis was performed as described (Engelowski et al., 2018). Data were analyzed pairwise Ba/F3-gp130-V_G_mIFNAR1-V_C_mIFNAR2 cells stimulated with 100 ng/ml GCCG versus without cytokine, stimulated with 100 U/ml IFNα4 versus without cytokine, and stimulated with 100 ng/ml GCCG versus 100 U/ml IFNα4. Transcriptome Analysis Console (TAC) software from Thermo Fisher Scientific was used for analysis.

### Statistical analyses

For proliferation assays, a representative experiment of *n* ≥ 3 assays with comparable results is displayed. IC_50_ values were calculated using a non-linear regression analysis in GraphPad Prism 6.1 (version 6.1 for Windows, GraphPad Software, La Jolla California United States). The data are presented as means ± S.D. For multiple comparisons, one-way ANOVA, followed by Bonferroni correction, was used (GraphPad Prism 6.1). Statistical significance was set at the level of *p* ≤ 0.05 (*p ≤ 0.05, **p ≤ 0.01, ***p ≤ 0.001).

### Data availability

The data of this study are available within the paper. Gene expression raw data have been deposited in the Gene Expression Omnibus (GEO) with the accession number GSE202839.

## Results

### Synthetic and natural cytokine receptors for type I interferons depend on Jak1 and Tyk2

**Figure 1:**
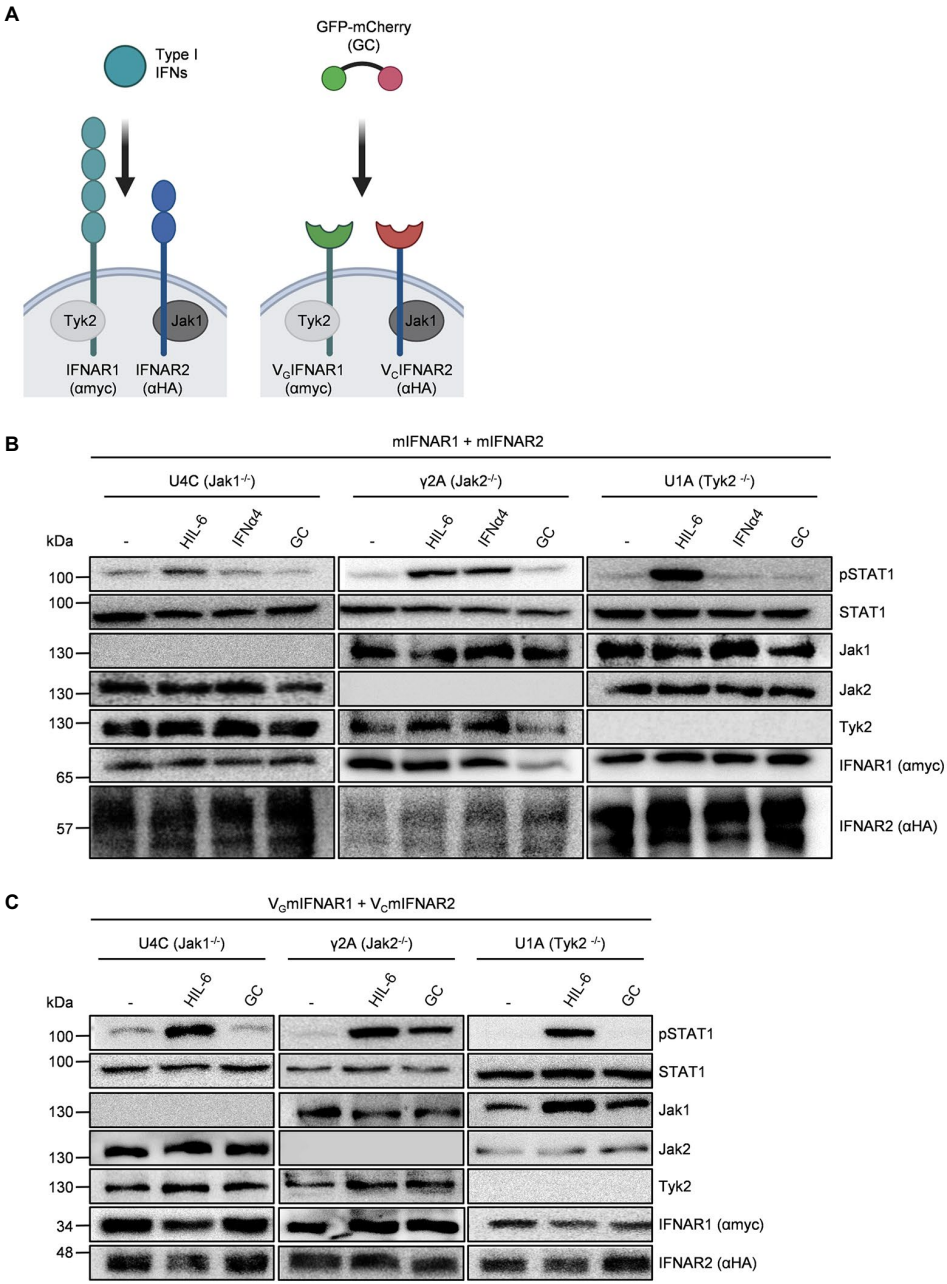
Native and synthetic type I IFN signaling depends on Jak1 and Tyk2. **(A)** Schematic overview of native and synthetic type I IFN signaling. Type I IFN signal *via* IFNAR1 (myc-tagged, lagoon) and IFNAR2 (HA tagged, blue) which are associated with Tyk2 and Jak1, respectively. In the synthetic receptors, the extracellular domains of IFNAR1 and IFNAR2 were replaced by nanobodies directed against GFP (green) and mCherry (red), respectively. Native IFNARs are activated by type I IFNs, while synthetic IFNARs were activated by dimeric GFP/mCherry ligands (GC). **(B)** STAT1 activation in U4C (Jak1^−/−^), γ2A (Jak2^−/−^) and U1A (Tyk2^−/−^) cells transiently expressing murine IFNAR1 and IFNAR2 treated with 200 U/ml IFNα4, 10 ng/ml Hyper-IL-6 or 10 ng/ml GC for 20 to 30 min. Equal amounts of proteins (50 μg/lane) were analyzed *via* specific antibodies detecting phospho-STAT1 and STAT1, Jak1, Jak2, Tyk2, IFNAR1 (myc-tagged), and IFNAR2 (HA-tagged). Western blot data shows one representative experiment out of three. **(C)** STAT1 activation in U4C (Jak1^−/−^), γ2A (Jak2^−/−^), and U1A (Tyk2^−/−^) cells transiently expressing murine V_G_IFNAR1 and V_C_IFNAR2 treated with 200 U/ml IFNα4, 10 ng/ml Hyper-IL-6 or 10 ng/ml GC for 20 to 30 min. Equal amounts of proteins (50 μg/lane) were analyzed *via* specific antibodies detecting phospho-STAT1 and STAT1, Jak1, Jak2, Tyk2, IFNAR1 (myc-tagged), and IFNAR2 (HA-tagged). Western blot data shows one representative experiment out of three.

Type I interferons resemble a large group of cytokines which all bind and signal *via* the IFNα/β receptor (IFNAR)1/2 (Figure 1A). Genetic exchange of the coding region for the extracellular domain of IFNAR1 by an anti-GFP nanobody (VHH_GFP_, V_G_) and of IFNAR2 by an anti-mCherry nanobody (VHH_mCherry_, V_C_) resulted in the synthetic cytokine receptors VHH_GFP_IFNAR1 (V_G_IFNAR1) and VHH_mCherry_IFNAR2 (V_C_IFNAR2). Whereas IFNAR1 and IFNAR2 are activated by type I interferons, V_G_IFNAR1 and V_C_IFNAR2 were expected to form functional receptor complexes after binding to the heterodimeric ligand GFP-mCherry (GC) fusion protein (Figure 1A). We used kinase-deficient U4C, γ2A and U1A cells transiently transfected with cDNAs coding for murine IFNAR1 and IFNAR2 to demonstrate the dependence of type I interferons on the receptor associated Janus kinases Tyk2 for IFNAR1 and Jak1 for IFNAR2. Deficiency of Jak1 in U4C, Jak2 in γ2A and Tyk2 in U1A cells was shown by Western blotting (Figures 1B,C). Expression of natural and synthetic mIFNAR1 and mIFNAR2 was also shown by Western blot detecting myc-tagged mIFNAR1 and HA-tagged mIFNAR2 (Figure 1B). Consequently, stimulation for 30 min with type I interferon IFNα4 (200 U/ml) induced STAT1 phosphorylation only in Jak2 deficient γ2A cells but not in Jak1 deficient U4C cells and Tyk2 deficient U1A cells (Figure 1B). Since all cells express the Jak1-dependent signal transducing Interleukin 6 receptor gp130, stimulation with 10 ng/ml of the IL-6/soluble IL-6R fusion protein Hyper-IL-6 (Fischer et al., 1997) for 20 min induced STAT1 phosphorylation in Jak2 deficient γ2A and Tyk2 deficient U1A but only marginally in Jak1 deficient U4C cells. Importantly, 10 ng/ml of the synthetic cytokine ligand GC did not induce signaling in any of the cell lines (Figure 1B). After transfection of U4C, γ2A and U1A cells with cDNAs coding for V_G_IFNAR1 and V_C_IFNAR2, only Jak2 deficient γ2A showed STAT1 phosphorylation after stimulation with 10 ng/ml of the synthetic cytokine GC fusion protein for 30 min but not the Jak1 deficient U4C and Tyk2 deficient U1A cells. These data indicate that synthetic IFNARs are biologically active and depend on the same Janus kinases as shown for the natural IFNARs.

### Synthetic IFNAR2 is biologically active as homodimer and heterodimer with IFNAR1

**Figure 2:**
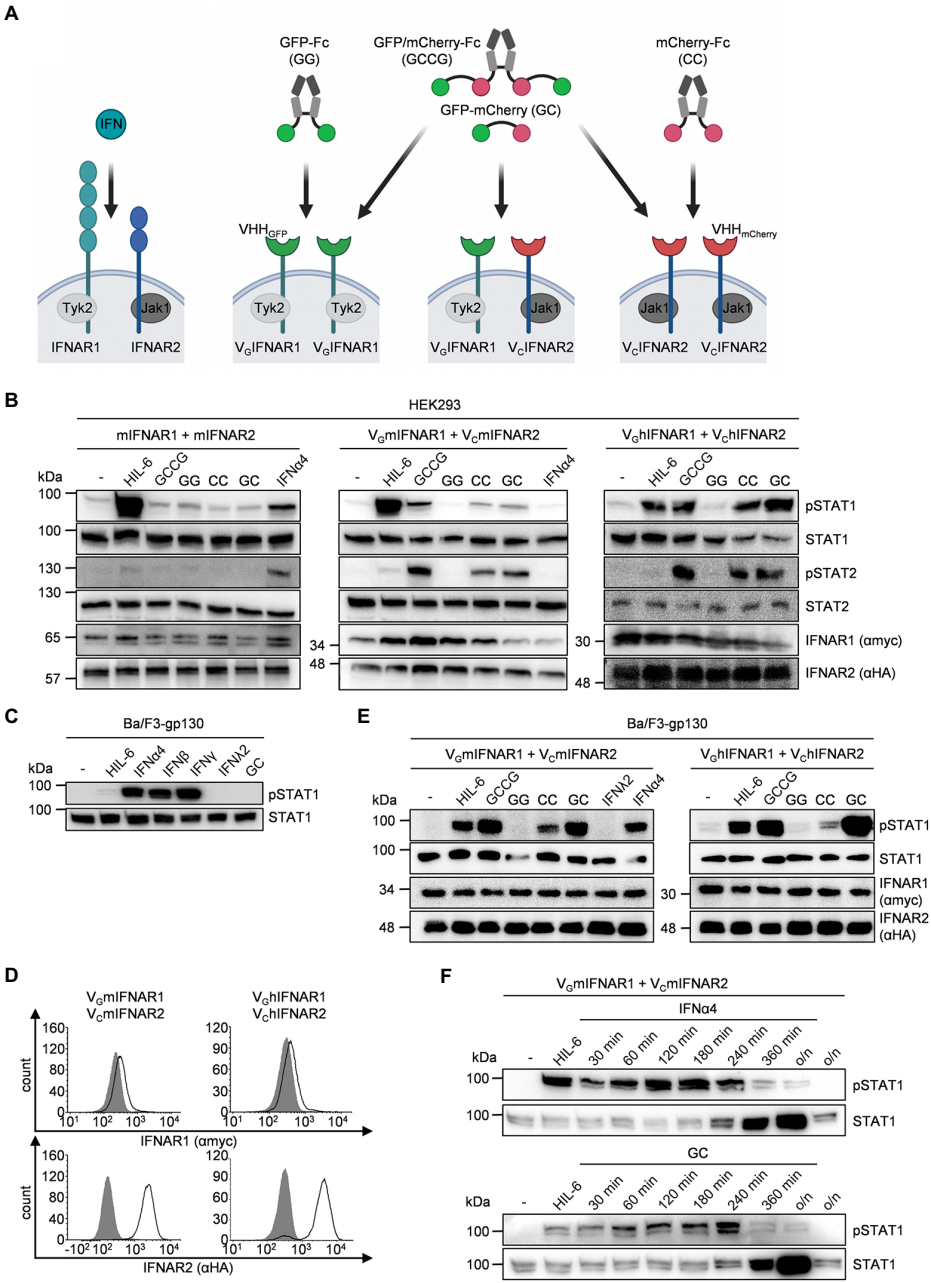
Comparable pattern and intensities of STAT activation by native and synthetic type I IFN receptors. **(A)** Whereas native type I IFN signaling depends on IFNAR1/2 heterodimers, the synthetic cytokines can engage different IFNAR1/2 receptor combinations. IFNAR1 (myc-tagged, lagoon) and IFNAR2 (HA-tagged, blue) which are associated with Tyk2 and Jak1, respectively. In the synthetic receptors, the extracellular domains of IFNAR1 and IFNAR2 were replaced by nanobodies directed against GFP (green) and mCherry (red), respectively. Native IFNARs are activated by type I IFNs, synthetic IFNARs were activated by multimeric GFP/mCherry ligands (GC, GG, CC, GCCG). **(B)** STAT1 and STAT2 activation in HEK293 cells transiently expressing murine IFNAR1 and IFNAR2 (left), murine V_G_IFNAR1 and V_C_IFNAR2 (middle) or human V_G_IFNAR1 and V_C_IFNAR2 (right) treated with 1,000 U/ml IFNα4, 10 ng/ml Hyper-IL-6 or 100 ng/ml of the synthetic cytokine ligands (GCCG, GG, CC, GC) for 30 min. Equal amounts of proteins (50 μg/lane) were analyzed *via* specific antibodies detecting phospho-STAT1, phospho-STAT2 and STAT1, STAT2, IFNAR1 (myc-tagged), and IFNAR2 (HA-tagged). Western blot data shows one representative experiment out of three. **(C)** STAT1 activation in Ba/F3-gp130 cells after stimulation with 200 U/ml IFNα4, 100 ng/ml IFNβ, 20 ng/ml IFNγ, 20 ng/ml IFNλ2, 10 ng/ml Hyper-IL-6, or 100 ng/ml of the synthetic cytokine ligands GC for 30 min. Equal amounts of proteins (50 μg/lane) were analyzed *via* specific antibodies detecting phospho-STAT1 and STAT1. Western blot data shows one representative experiment out of three. **(D)** Cell surface expression of Ba/F3-gp130 cells transfected with either murine or human V_G_IFNAR1 and V_C_IFNAR2. Expression was detected *via* antibodies detecting myc-tagged IFNAR1 (upper panel) or HA-tagged IFNAR2 (lower panel) and is indicated as solid line. Gray-shade areas indicate non transfected Ba/F3-gp130 cells (negative control). **(E)** STAT1 activation in Ba/F3-gp130 cells stably expressing murine V_G_IFNAR1 and V_C_IFNAR2 (left) or human V_G_IFNAR1 and V_C_IFNAR2 (right) treated with 10 ng/ml Hyper-IL-6, 100 ng/ml of the synthetic cytokine ligands (GCCG, GG, CC, GC), 200U/ml IFNα4 or 20 ng/ml IFNλ2 for 30 min. Equal amounts of proteins (50 μg/lane) were analyzed *via* specific antibodies detecting phospho-STAT1, STAT1, IFNAR1, and IFNAR2. Western blot data shows one representative experiment out of three. **(F)** STAT1 activation in Ba/F3-gp130 cells stably expressing murine V_G_IFNAR1 and V_C_IFNAR2 treated with 10 ng/ml Hyper-IL-6 (30 min) and 200 U/ml IFNα4 or 100 ng/ml of the synthetic cytokine ligand GC for the indicated time points. Equal amounts of proteins (50 μg/lane) were analyzed *via* specific antibodies detecting phospho-STAT1 and STAT1. Western blot data shows one representative experiment out of three.

Next, we used human (HEK293) and murine cells (Ba/F3) to verify functional heterodimeric receptor complex formation of V_G_mIFNAR1 and V_C_mIFNAR2 by dimeric GC and tetrameric GCCG synthetic ligands (Figure 2A). Moreover, we analyzed IFNAR1 and IFNAR2 homodimerization using homodimeric synthetic ligands GG and CC. As described previously, GG, CC, and GCCG are based on dimeric IgG1 Fc-fusion proteins G-Fc, C-Fc, and GC-Fc, respectively (Mossner et al., 2020). As shown in Figure 2B, phosphorylation of STAT1 and STAT2 in HEK293 cells transiently transfected with cDNAs coding for mIFNAR1 and mIFNAR2 was achieved after stimulation with 1,000 U/ml IFNα4 but not after stimulation with 100 ng/ml GCCG, GG, CC, and GC for 30 min. As expected, stimulation with 10 ng/ml Hyper-IL-6 (HIL-6) for 30 min induced STAT1 but not STAT2 phosphorylation (Fischer et al., 1997). HEK293 cells transiently transfected with cDNAs coding for murine or human V_G_IFNAR1 and V_C_IFNAR2 were also stimulated with 100 ng/ml GCCG, GG, CC, GC, and Hyper-IL-6 for 30 min. As seen for IFNα4, GC, and GCCG induced STAT1 and STAT2 phosphorylation in HEK293 cells expressing murine or human V_G_IFNAR1 and V_C_IFNAR2. Interestingly, stimulation with 100 ng/ml CC but not with 100 ng/ml GG induced a similar but slightly weaker STAT1 and STAT2 phosphorylation pattern suggesting that homodimeric murine and human V_C_IFNAR2s were biologically active, whereas homodimeric murine and human V_G_IFNAR1 did not result in STAT1 and STAT2 phosphorylation (Figure 2B).

Next, we used the IL-6/sIL-6R dependent murine pre-B cell line Ba/F3-gp130. We stimulated Ba/F3-gp130 cells with the type I interferons IFNα4 and IFNβ, the type II interferon IFNγ and the type III interferon IFNλ2. As shown before type I IFN induced STAT1 phosphorylation (Jaster et al., 1997). Interestingly, also type II interferons but not type III interferons or GC induced STAT phosphorylation (Figure 2C). Next, Ba/F3-gp130 cells were stably transduced with cDNAs coding for murine or human V_G_IFNAR1 and V_C_IFNAR2, and cell-surface expression was analyzed by flow cytometry (Figure 2D). Stimulation of Ba/F3-gp130-V_G_IFNAR1-V_C_IFNAR2 cells with 100 ng/ml synthetic cytokine ligands for 30 min revealed that GC and GCCG also induced type I interferon-like STAT1 phosphorylation, whereas STAT1 phosphorylation by CC-induced homodimeric V_C_IFNAR2 was less intense (Figure 2E). A time course experiment was carried out to investigate the dynamics of STAT1 phosphorylation in Ba/F3-gp130-V_G_mIFNAR1-V_C_mIFNAR2 cells stimulated with IFNα4 or GC. STAT1 phosphorylation was analyzed by Western blotting at the indicated time points (Figure 2F). Interestingly, STAT1 expression in Ba/F3-gp130-V_G_mIFNAR1-V_C_mIFNAR2 cells increased 240 min after stimulation with either IFNα4 or GC.

**Figure 3:**
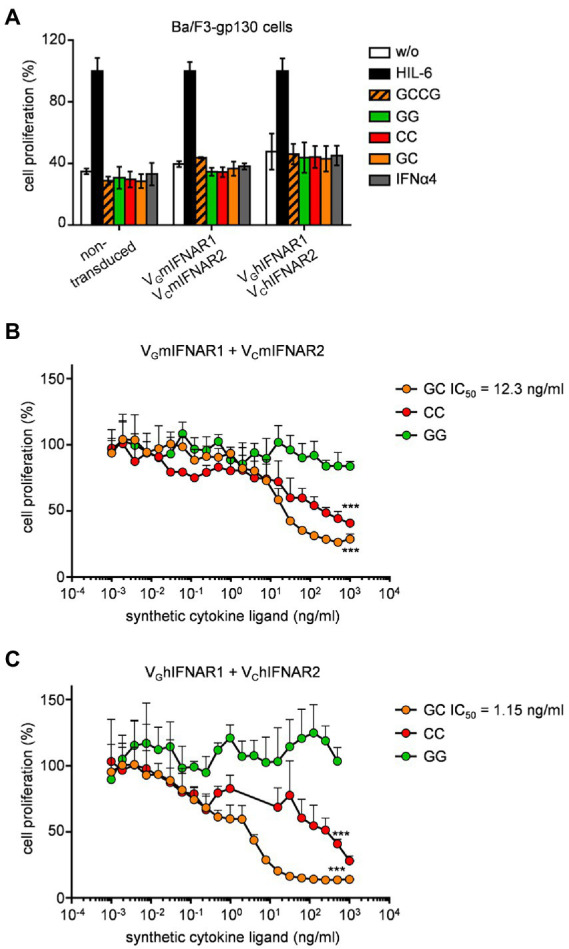
Synthetic type I IFN signaling inhibits Hyper-IL-6 induced proliferation of Ba/F3-gp130 cells. **(A)** Proliferation of Ba/F3-gp130, Ba/F3-gp130-V_G_mIFNAR1-V_C_mIFNAR2, and Ba/F3-gp130-V_G_hIFNAR1-V_C_hIFNAR2 cells without cytokine (−), with 10 ng/ml Hyper-IL-6, 100 ng/ml GCCG, GG, CC, GC or 200 U/ml IFNα4. One representative experiment out of three is shown. **(B)** Proliferation of Ba/F3-gp130-V_G_mIFNAR1-V_C_mIFNAR2 cells with 10 ng/ml Hyper-IL-6 in the presence of increasing concentrations of GG, CC, and GC (0.001–1,000 ng/ml). One representative experiment out of three is shown. **(C)** Proliferation of Ba/F3-gp130-V_G_hIFNAR1-V_C_hIFNAR2 cells with 10 ng/ml Hyper-IL-6 in the presence of increasing concentrations of GG, CC, GC (0.001–1,000 ng/ml). One representative experiment out of three is shown. ***p ≤ 0.001.

Proliferation of Ba/F3-gp130 is dependent on Hyper-IL-6 and mediated by STAT3 and ERK phosphorylation (Fischer et al., 1997; Figure 3A). Stimulation of Ba/F3-gp130 cells with 200 U/ml type I interferon failed to induce cellular proliferation, which was also observed for Ba/F3-gp130-V_G_IFNAR1-V_C_IFNAR1 cells after stimulation with 1,000 ng/ml synthetic cytokine ligands GCCG, GG, CC, and GC (Figure 3A). It was, however, previously shown that type I IFN signaling blocks IL-3-induced cellular proliferation of Ba/F3 cells (Jaster et al., 1999). Ba/F3-gp130 cells expressing murine or human V_G_IFNAR1 and V_C_IFNAR2 were stimulated with 10 ng/ml Hyper-IL-6, which induce cellular proliferation and was defined as 100% proliferation (Figures 3B,C). Simultaneous titration of increasing amounts of GC, CC and GG into Hyper-IL-6 stimulated Ba/F3 cell lines resulted in dose-dependent inhibition of cellular proliferation for GC and CC which induced murine or human V_G_IFNAR1 and V_C_IFNAR2 heterodimers and murine or human V_C_IFNAR2 homodimers, respectively. GG induced IFNAR1 homodimers had no effect. Half maximal inhibitory concentrations (IC_50_) were calculated for GC for murine synthetic IFNARs to be 12.3 ng/ml, respectively (Figure 3B) and for human synthetic IFNARs 1.15 ng/ml (Figure 3C).

**Figure 4:**
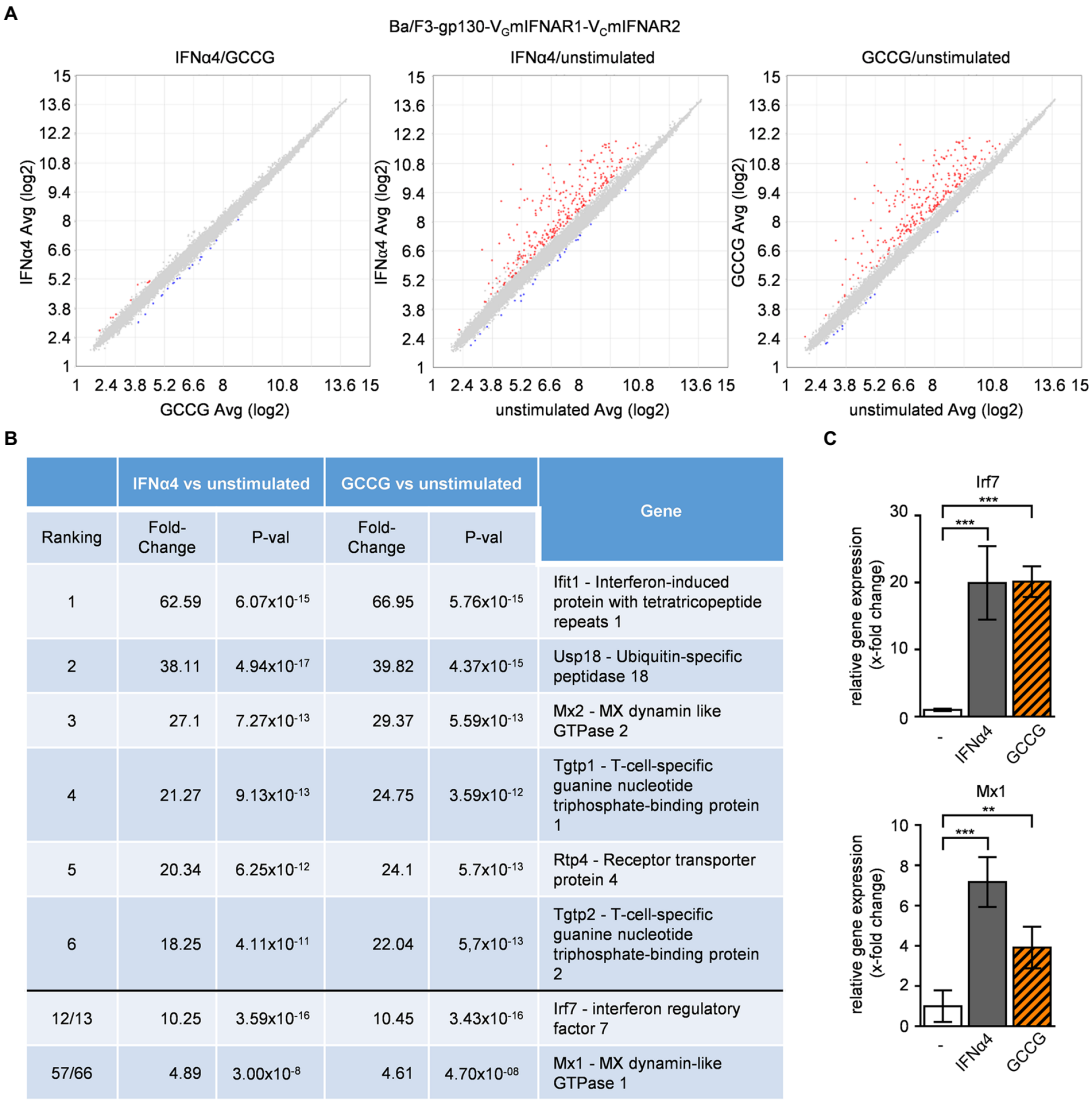
Comparable gene regulation pattern by native and synthetic type I IFN receptors. Microarray analysis of Ba/F3-gp130-V_G_mIFNAR1-V_C_mIFNAR2 cells either stimulated with IFNα4 or GCCG. The comparison was performed with 1.5-fold (*p*-value < 0.05). Varying upregulated genes are shown in red, downregulated genes in blue. **(A)** Scatter blot comparing mRNA levels of IFNα4 (200 U/ml) and GCCG (100 ng/ml) stimulation of Ba/F3-gp130-V_G_mIFNAR1-V_C_mIFNAR2 cells; left: IFNα4 vs. GCCG, middle: IFNα4 vs. unstimulated, right: GCCG vs. unstimulated. Cells were stimulated for 240 min. **(B)** List of regulated mRNAs for IFNα4 or GCCG stimulation with respective fold change and p-value. **(C)** Verification of microarray data by quantification of mRNA expression of two genes (Irf7, Mx1) for IFNα4 or GCCG stimulation of Ba/F3-gp130-V_G_mIFNAR1-V_C_mIFNAR2 cells. Mean value of four independent experiments is shown. ^**^p ≤ 0.01; ^***^p ≤ 0.001.

Next, we analyzed the mRNA-expression by gene-array analysis of Ba/F3-gp130-V_G_mIFNAR1-V_C_mIFNAR2 cells stimulated with 200 U/ml IFNα4 and 100 ng/ml GCCG for 240 min, which indicated a high (99.91%) overlap of overall gene regulation (Figure 4A). The regulated genes are typical type I IFN target genes, including Ifit1, Usp18, Mx1 and Mx2 and Irf7 (Figure 4B). mRNA-levels of Irf7 and Mx1 were independently verified by qPCR (Figure 4C) supporting our data obtained from the gene-array analysis. This indicates that the synthetic heterodimeric IFNARs induced the same biological outcome as the natural heterodimeric IFNARs.

### Synthetic IFNARs have antiviral activity

**Figure 5:**
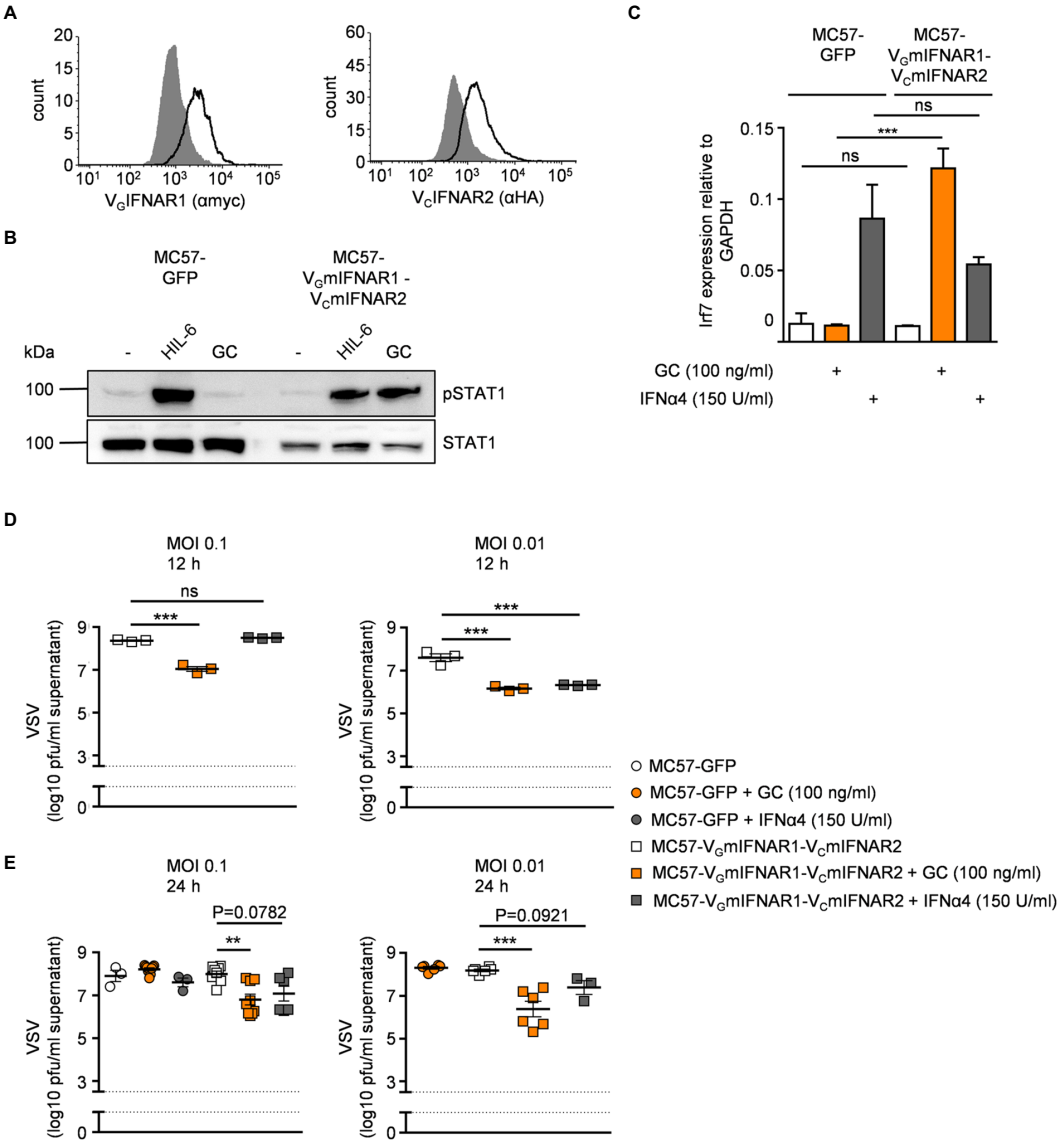
Synthetic IFNARs have antiviral activity in MC57 cells. **(A)** Cell surface expression of MC57 cells transduced with murine V_G_IFNAR1 and V_C_IFNAR2. Expression was demonstrated *via* antibodies detecting myc-tagged IFNAR1 (left panel) or HA-tagged IFNAR2 (right panel) and is indicated as solid line. Gray-shade areas indicate non transduced MC57 cells (negative control). **(B)** STAT1 activation in MC57 cells stably expressing murine V_G_IFNAR1 and V_C_IFNAR2 treated with 10 ng/ml Hyper-IL-6 or 100 ng/ml of the synthetic cytokine ligand GC for 30 min. Equal amounts of proteins (50 μg/lane) were analyzed *via* specific antibodies detecting phospho-STAT1, STAT1. Western blot data shows one representative experiment out of three. **(C)** Quantification of mRNA expression of Irf7 for IFNα4 (150 U/ml) or GC (100 ng/ml) stimulation of MC57-GFP and MC57-V_G_mIFNAR1-Vm_C_IFNAR2 cells. One representative experiment out of three is shown. **(D)** MC57-V_G_mIFNAR1-Vm_C_IFNAR2 cells were treated with medium, IFNα4 (150 U/ml) or synthetic GC ligand (100 ng/ml), followed by infection with VSV (multiplicity of infection [MOI]). Viral titers in the supernatants were measured 12 h after infection. **(E)** MC57-GFP and MC57-V_G_mIFNAR1-Vm_C_IFNAR2 cells were treated with medium, IFNα4 (150 U/ml) or synthetic GC ligand (100 ng/ml), followed by infection with VSV (multiplicity of infection [MOI]). Viral titers in the supernatants were measured 24 h after infection. **p ≤ 0.01; ***p ≤ 0.001.

The mouse fibroblast cell line MC57 is used for *in vitro* infection assays with vesicular stomatitis virus (VSV; Lang et al., 2009). Stimulation of MC57 cells with type I IFNs significantly reduced VSV load as determined by plaque assays (Lang et al., 2009). Here, MC57 cells were stably transduced with V_G_mIFNAR1 and V_C_mIFNAR2 and cell surface expression was detected by flow cytometry (Figure 5A). Stimulation with 100 ng/ml GC for 30 min induced STAT1 phosphorylation in MC57-V_G_mIFNAR1-V_C_mIFNAR2 cells but not in MC57 control cells transduced with GFP (Figure 5B). Type I IFN-like Irf7 gene expression was also inducible in MC57 cells expressing V_G_mIFNAR1-V_C_mIFNAR2 but not in GFP expressing MC57 cells after stimulation with GC (Figure 5C). MC57-V_G_mIFNAR1-V_C_mIFNAR2 and MC57-GFP cells were infected with VSV (multiplicity of infection [MOI] = 0.1 and 0.01). Cells were pre-incubated for 120 min with 100 ng/ml GC, IFNα4 (150 U/ml) or left untreated. GC and IFNα4 reduced viral load after infection in MC57-V_G_mIFNAR1-V_C_mIFNAR2 cells compared to the untreated control and GC-treated MC57-GFP cells (12 and 24 h time points in Figures 5D,E). These data showed that synthetic IFNARs have antiviral activity.

### Deletion variants for synthetic IFNARs assign a major role for Y510 in murine IFNAR2 for STAT1 and STAT2 phosphorylation

**Figure 6:**
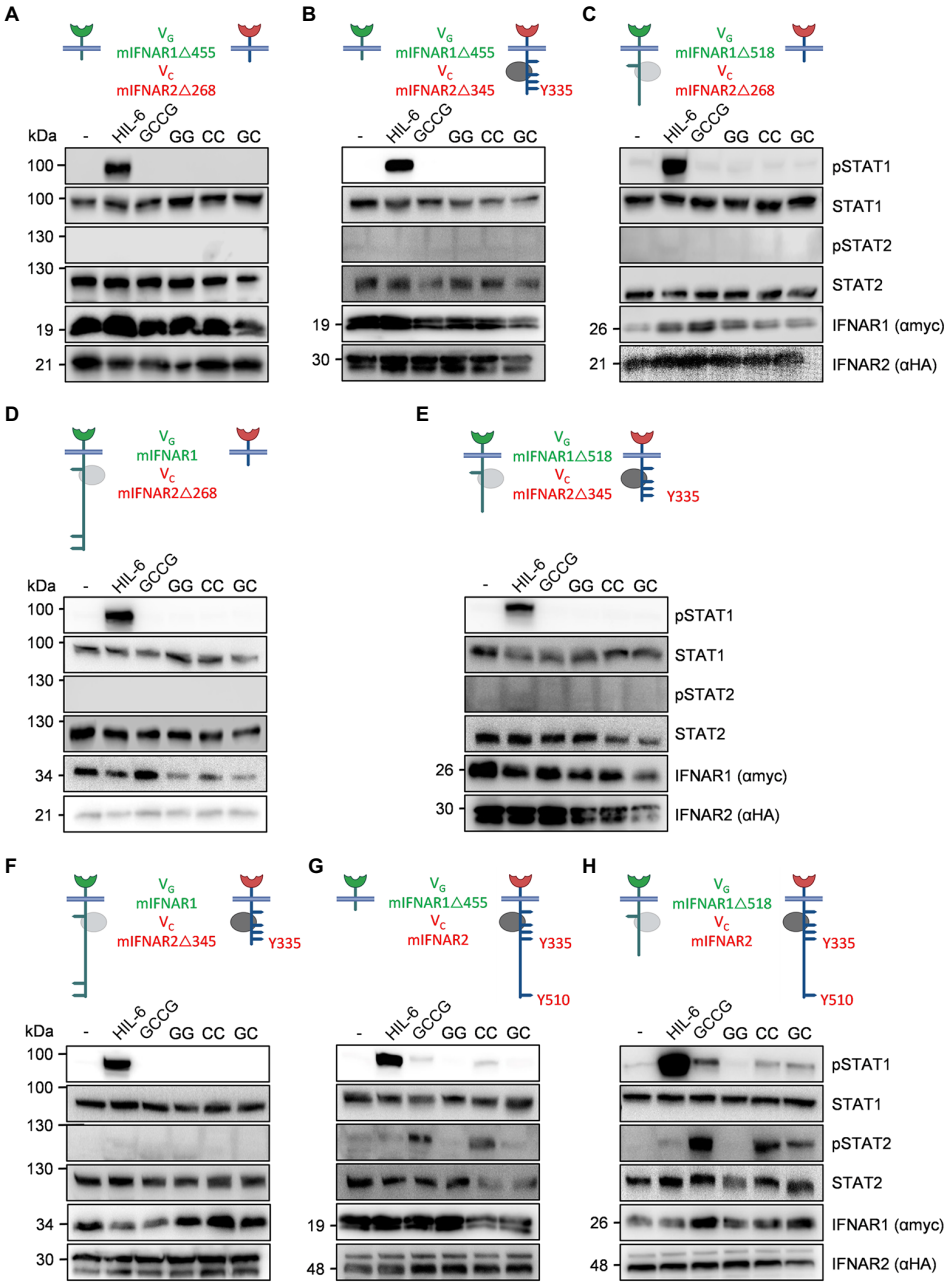
Deletion variants for synthetic murine IFNARs highlight the importance of Y510 in IFNAR2. The following murine receptor combinations were transiently transfected into HEK293 cells: **(A)** V_G_IFNAR1Δ455 and V_C_IFNAR2Δ268, **(B)** V_G_IFNAR1Δ455 and V_C_IFNAR2Δ345, **(C)** V_G_IFNAR1Δ518 and V_C_IFNAR2Δ268, **(D)** V_G_IFNAR1 and V_C_IFNAR2Δ268, **(E)** V_G_IFNAR1Δ518 and V_C_IFNAR2Δ345, **(F)** V_G_IFNAR1 and V_C_IFNAR2Δ345, **(G)** V_G_IFNAR1Δ455 and V_C_IFNAR2, and **(H)** V_G_IFNAR1Δ518 and V_C_IFNAR2. Conserved tyrosine residues between human and murine receptors are indicated. **(A–H)** STAT1 and STAT2 activation was determined in transiently transfected HEK293 cells treated with 10 ng/ml Hyper-IL-6, 100 ng/ml of the synthetic cytokine ligands (GCCG, GG, CC, GC) for 30 min. Equal amounts of proteins (50 μg/lane) were analyzed *via* specific antibodies detecting phospho-STAT1, phospho-STAT2, STAT1, STAT2, IFNAR1 (myc-tagged), and IFNAR2 (HA-tagged). Western blot data shows one representative experiment out of three.

In mice, the intracellular domain of IFNAR1 has four (455, 518, 529, 576) tyrosine residues and four in humans (466, 481, 527, 538). Murine IFNAR2 has six (268, 315, 317, 335, 398, 510) and human IFNAR2 seven intracellular tyrosine residues (269, 306, 316, 318, 337, 411, 512), which in both receptors might serve as STAT binding sites (Table 1), albeit the tyrosines are not imbedded in classical SH2 domain binding sites [pYxxP for STAT1, pYxxQ for STAT3, pYxxL for STAT5, pYxxF for STAT6 (Morris et al., 2018)]. However, some binding site deviations and tyrosine-independent mechanisms were described (Puigdevall et al., 2022). Previous work suggested that only the tyrosine residues in human IFNAR2 are important for STAT1/2 activation (Shemesh et al., 2021). Here, we used the synthetic IFNARs to reassign tyrosine residues as STAT activation sites. First of all, we entirely deleted the intracellular domain including Janus kinase binding sites in V_G_mIFNAR1Δ455 and V_C_mIFNAR2Δ268. As expected, co-expression of V_G_mIFNAR1Δ455 with V_C_mIFNAR2Δ268 in HEK293 cells failed to induce STAT1/2 phosphorylation after stimulation with 100 ng/ml GCCG, GG, CC and GC for 30 min (Figure 6A). The variants V_G_mIFNAR1Δ518 and V_C_mIFNAR2Δ345 contain Janus kinase Box1 and Box2 binding motifs. Moreover, they include tyrosine residues Y455 but lacked Y518, Y529 and Y576 in murine IFNAR1, and include Y268, Y315, Y317, Y335 but lacked Y398 and Y510 in murine IFNAR2. No activation with any of the synthetic cytokines was, however, observed after co-expression of V_G_mIFNAR1Δ455 (no Tyk2 binding)/V_C_mIFNAR2Δ345 (Jak1 binding, but no conserved Y510; Figure 6B), V_G_mIFNAR1Δ518 (Tyk2 binding, lacking conserved Y518/529)/V_C_mIFNAR2Δ268 (no Jak1 binding; Figure 6C), V_G_mIFNAR1/V_C_mIFNAR2Δ268 (no Jak1 binding; Figure 6D), V_G_mIFNAR1Δ518 (Tyk2 binding, lacking conserved Y518/529)/V_C_mIFNAR2Δ345 (Jak1 binding, no conserved Y510; Figure 6E) and V_G_mIFNAR1/V_C_mIFNAR2Δ345 (Jak1 binding, no conserved Y510; Figure 6F). These experiments suggested that apart from Jak binding site(s), Y510 in murine IFNAR2 is crucial for STAT1 and STAT2 activation.

**Table 1:** Overview about amino acid surrounding tyrosine residues.

IFNAR1		IFNAR2	
Murine	Human	Murine	Human
VWK**Y**_455_LCH	CIN**Y**_466_VFF	RIG**Y**_268_ICL	WIG**Y**_269_ICL
	IDE**Y**_481_FSE		EVI**Y**_306_INR
LRK**Y**_518_SSQ	HKK**Y**_527_SSQ	LWN**Y**_315_D**Y**_317_EDG	VWD**Y**_316_N**Y**_318_DDE
SGN**Y**_529_SNE	SGN**Y**_538_SNE	VTG**Y**_335_TMH	GGG**Y**_337_TMH
NEK**Y**_576_LQS		TGP**Y**_398_ERR	
			EED**Y**_411_SST
		GDG**Y**_510_IMR	GDG**Y**_512_IMR

Classical SH2 domain binding sites pYxxP for STAT1, pYxxQ for STAT3, pYxxL for STAT5, pYxxF for STAT6. Tyrosine residues are highlighted in bold.

Next, V_G_mIFNAR1Δ455 (no Tyk2 binding)/V_C_mIFNAR2 showed STAT1 and STAT2 phosphorylation only after stimulation with GCCG and CC which point to functional homodimerization of full-length V_C_mIFNAR2 (Figure 6G). Finally, we combined V_G_mIFNAR1Δ518 (Tyk2 binding, lacking conserved Y518/529) and V_C_mIFNAR2 which resulted in STAT1 and STAT2 phosphorylation after stimulation with GCCG, CC and GC (Figure 6H). As seen before, GG stimulation did not result in STAT1 and STAT2 phosphorylation for any IFNAR1 combination (Figure 6). Our data showed that homodimeric activation of IFNAR2 results in STAT1/2 phosphorylation only for the full-length synthetic receptor. For heterodimeric IFNAR-induced STAT1/2 phosphorylation, binding of Tyk2 to IFNAR1 and the complete intracellular domain of IFNAR2 were needed. Taken together, the tyrosine residues in the intracellular domain of IFNAR1 did not contribute to STAT1/2 phosphorylation and Y510 in IFNAR2 appears to be critical for STAT1/2 phosphorylation.

### Mutational analysis assigned a minor role for Y335 and a major role for Y510 in murine IFNAR2 for STAT1 and STAT2 phosphorylation

**Figure 7:**
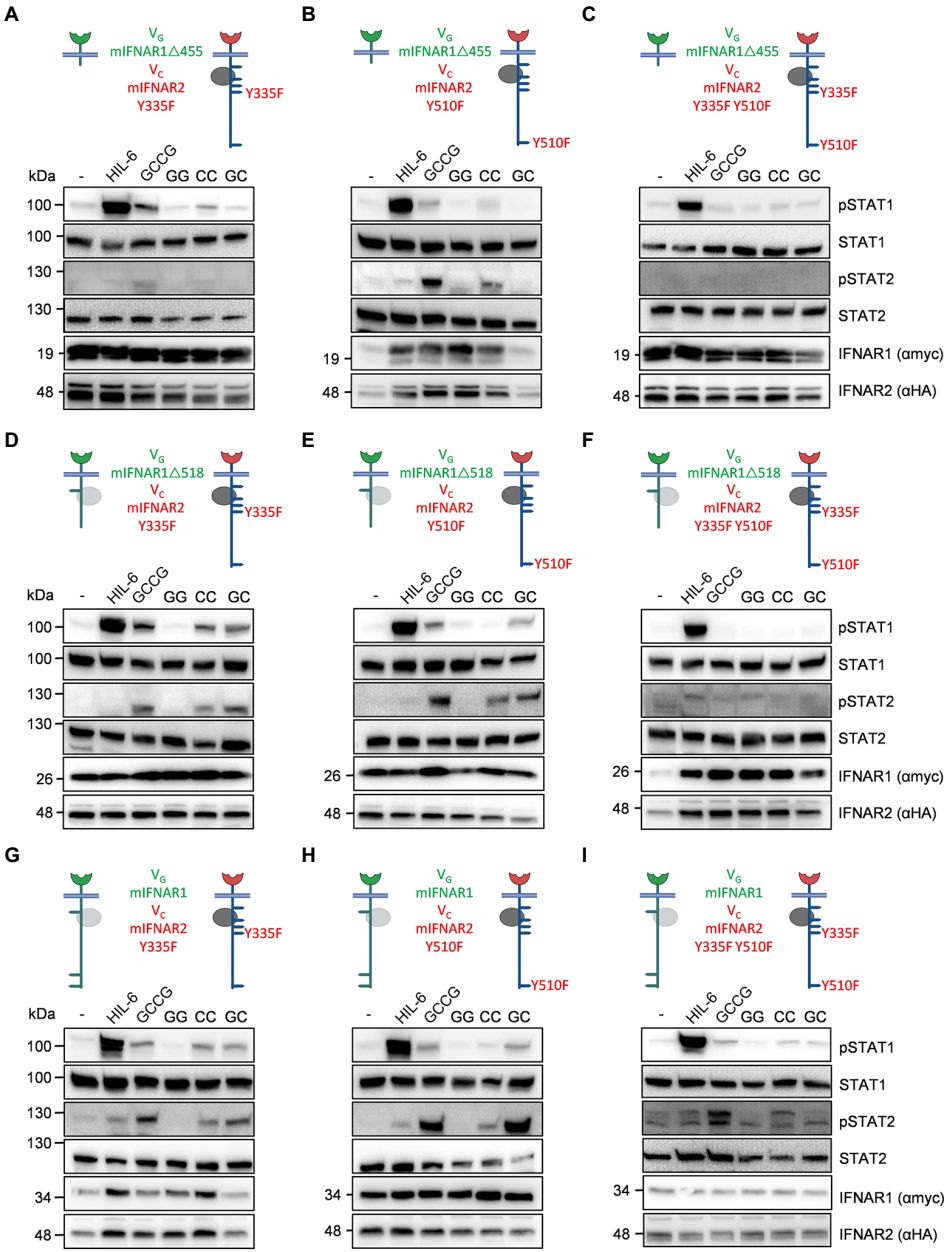
Point mutation variants for synthetic murine IFNARs support the importance of Y510 in IFNAR2. The following murine receptor combinations were transiently transfected into HEK293 cells: **(A)** V_G_IFNAR1Δ455 and V_C_IFNAR2_Y335F, **(B)** V_G_IFNAR1Δ455 and V_C_IFNAR2_Y510F, **(C)** V_G_IFNAR1Δ455 and V_C_IFNAR2_Y335F_Y510F, **(D)** V_G_IFNAR1Δ518 and V_C_IFNAR2_Y335F, **(E)** V_G_IFNAR1Δ518 and V_C_IFNAR2_Y510F, **(F)** V_G_IFNAR1Δ518 and V_C_IFNAR2_Y335F_Y510F, **(G)** V_G_IFNAR1 and V_C_IFNAR2_Y335F, **(H)** V_G_IFNAR1 and V_C_IFNAR2_Y510F, and **(I)** V_G_IFNAR1 and V_C_IFNAR2_Y335F_Y510F. Conserved tyrosine residues between human and murine receptors are indicated. (**A–H**) STAT1 and STAT2 activation was determined in transiently transfected HEK293 cells treated with 10 ng/ml Hyper-IL-6, 100 ng/ml of the synthetic cytokine ligands (GCCG, GG, CC, GC) for 30 min. Equal amounts of proteins (50 μg/lane) were analyzed *via* specific antibodies detecting phospho-STAT1, phospho-STAT2, STAT1, STAT2, IFNAR1 (myc-tagged), and IFNAR2 (HA-tagged). Western blot data shows one representative experiment out of three.

To independently define the tyrosine residues, responsible for STAT1 and STAT2 phosphorylation in type I IFN signaling, we decided to mutate Y335 and Y510 in mIFNAR2 into F335 and F510. Albeit Y335 appears not to be involved in STAT1/2 phosphorylation as suggested by IFNAR2 deletion variant V_C_mIFNAR2Δ345 (see Figure 6), we cannot exclude that the STAT binding site at Y335 might simply be disturbed by the nearby deletion at position 345. Single and double tyrosine (Y) to phenylalanine (F) mutants at position 335 and 510 of V_C_mIFNAR2 were combined with V_G_mIFNAR1Δ455 (no Tyk2 binding), V_G_mIFNAR1Δ518 (Tyk2 binding, lacking conserved Y518/529) or full-length V_G_mIFNAR1 in HEK293 cells (Figure 7). Stimulation of transiently transfected HEK293 cells with cDNAs coding for V_G_mIFNAR1Δ455 (no Tyk2 binding) and V_C_mIFNAR2_Y335F, V_C_mIFNAR2_Y510F or V_C_mIFNAR2_Y335F_Y510F with 100 ng/ml GCCG, GG, CC, and GC for 30 min showed that STAT1 and STAT2 phosphorylation was only detectable after homodimeric activation of V_C_mIFNAR2_Y335F and V_C_mIFNAR2_Y510F but not of V_C_mIFNAR2_Y335F_Y510F by GCCG and CC (Figures 7A–C). Next, the cDNA coding for V_G_mIFNAR1Δ518 (Tyk2 binding, lacking conserved Y518/529) was transiently transfected into HEK293 cells together with cDNAs coding for V_C_mIFNAR2_Y335F, V_C_mIFNAR2_Y510F or V_C_mIFNAR2_Y335F_Y510F and stimulated with 100 ng/ml GCCG, GG, CC, and GC for 30 min (Figures 7D–F). Here, heterodimeric receptor activation *via* GC and GCCG was detected for V_G_mIFNAR1Δ518 with V_C_mIFNAR2_Y335F and V_C_mIFNAR2_Y510F but not with V_C_mIFNAR2_Y335F_Y510F. The same pattern of STAT1/2 phosphorylation in HEK293 cells was seen for the combination of full length V_G_mIFNAR1 with V_C_mIFNAR2_Y335F, V_C_mIFNAR2_Y510F or V_C_mIFNAR2_Y335F_Y510F after stimulation with GCCG, GG, CC, and GC (Figures 7G–I). Albeit on different blots, homodimeric stimulation V_C_mIFNAR2_Y335F resulted in slightly stronger STAT1/2 phosphorylation compared to V_C_mIFNAR2_Y510F (compare CC stimulation of Figures 7A,D,G with CC stimulation of Figures 7B,E,H), suggesting that Y510 is more important as Y335. Taken together, our data indicated that IFNAR1 did not directly contribute to STAT1 and STAT2 phosphorylation *via* direct STAT binding sites. For IFNAR2, Y510 has a stronger impact on STAT1 and STAT2 phosphorylation as Y335, which only played a minor role.

### Human IFNAR2 is critical for STAT1 and STAT2 phosphorylation

**Figure 8:**
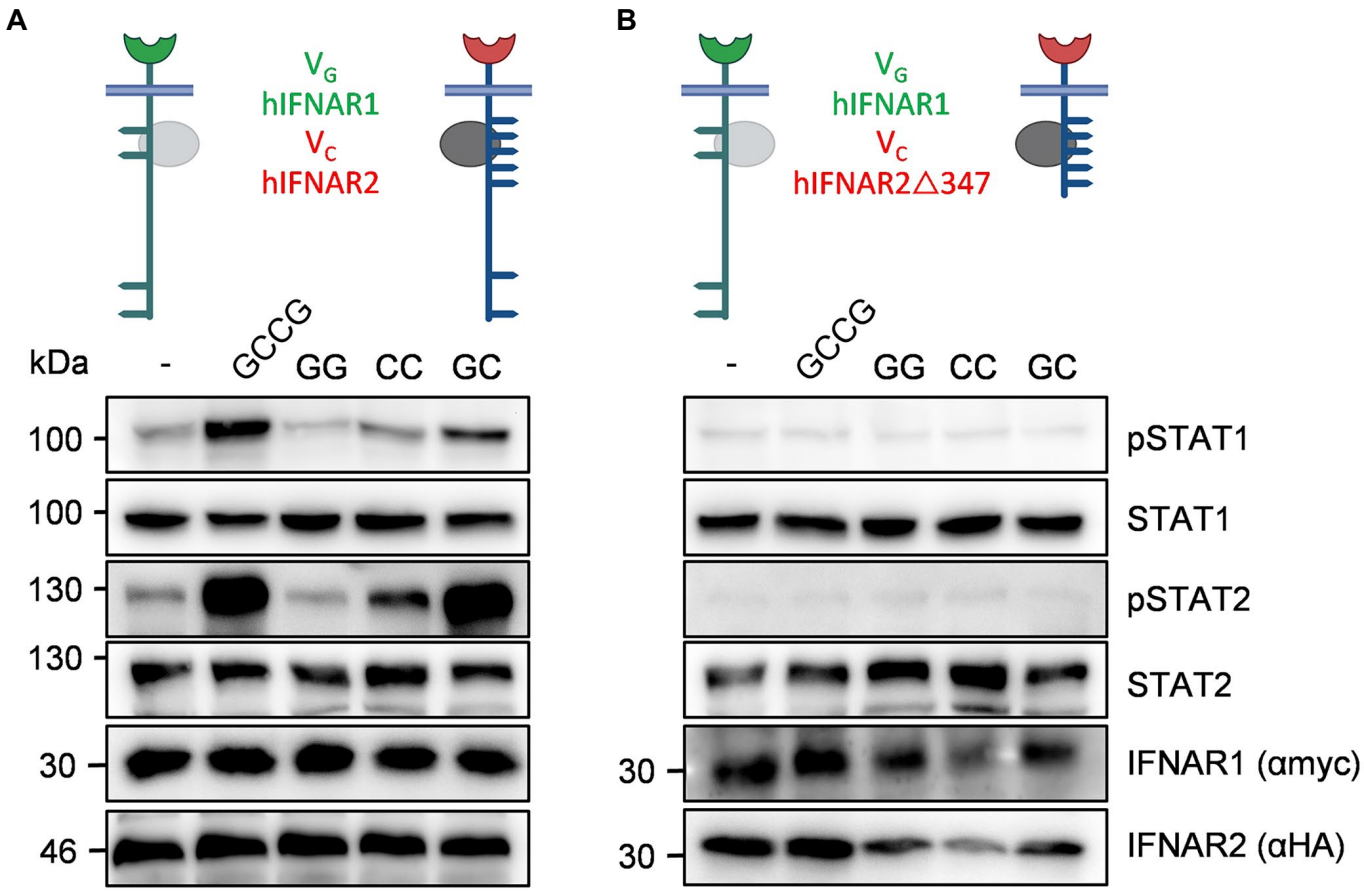
Human IFNAR2 is critical for STAT1 and STAT2 phosphorylation. The following human receptor combinations were transiently transfected into HEK293 cells: **(A)** V_G_IFNAR1 and V_C_IFNAR2 and **(B)** V_G_IFNAR1 and V_C_IFNAR2Δ347. **(A,B)** STAT1 and STAT2 activation was determined in transiently transfected HEK293 cells treated with 10 ng/ml Hyper-IL-6, 100 ng/ml of the synthetic cytokine ligands (GCCG, GG, CC, GC) for 30 min. Equal amounts of proteins (50 μg/lane) were analyzed *via* specific antibodies detecting phospho-STAT1, phospho-STAT2, STAT1, STAT2, IFNAR1 (myc-tagged), and IFNAR2 (HA-tagged). Western blot data shows one representative experiment out of three.

Finally, we analyzed STAT1 and STAT2 phosphorylation in the synthetic human IFNARs. cDNAs coding for V_G_hIFNAR1 and V_C_hIFNAR2 or V_C_hIFNAR2Δ347 were transiently transfected into HEK293 cells. V_C_hIFNAR1Δ347 (Jak1 binding, no Y411, Y512) is the equivalent to V_C_mIFNAR2Δ345 (Jak1 binding, no Y510). HEK293 cells, transfected with cDNAs coding for V_G_hIFNAR1 and V_C_hIFNAR2 showed sustained heterodimeric IFNAR1/2 and slight homodimeric IFNAR2 receptor activation of STAT1 and STAT2 phosphorylation after stimulation with the synthetic cytokine ligands GCCG, CC and GC but not with GG (100 ng/ml; Figure 8A). HEK293 cells expressing V_G_hIFNAR1 and V_C_hIFNAR2Δ347, however, failed to induce STAT1 and STAT2 phosphorylation after stimulation with GCCG, CC or GC, indicating that also human IFNAR2 carry the STAT1/2 binding sites, with a major involvement of Y512 (Figure 8B). Surprisingly, Y512 which is the homologue residue to Y510 in murine IFNAR2 is not embedded in a typical STAT binding site (Puigdevall et al., 2022). We cannot, however, exclude an involvement of Y411. Alternatively, the missing STAT activation might be due to the deletion of the constitutive binding site in IFNAR2Δ347 (Shemesh et al., 2021).

## Discussion

We embedded the type I IFN receptors IFNAR1 and IFNAR2 into a fully synthetic receptor/ligand system, which was achieved by replacing the extracellular domain of the IFNARs by nanobodies directed against GFP and mCherry. To initiate synthetic type I IFN signaling, we used heteromeric GFP-mCherry fusion proteins with high specificity and affinity. Synthetic IFNARs behave like their natural counterparts with respect to Jak activation, STAT1/2 phosphorylation and transcriptomic regulation. We focused on STAT1 and STAT2 phosphorylation as read-out of synthetic IFNAR activation because these are characteristic for type I IFNs. However, we are aware that overall signal transduction of type I IFNs is much more complex (Puigdevall et al., 2022). Since we did not modify the transmembrane and intracellular domain of IFNAR1 and IFNAR2 and also performed in depth transcriptomic analysis, it is, however, very likely that the replacement of the extracellular domains did not change signaling of the synthetic vs. natural receptors.

We used IFNα4 to compare signaling of natural IFNARs with GC-induced signaling of synthetic IFNARs. We chose IFNα4 and not IFNα2, which is the predominantly type I IFN used in the clinic (Pestka, 2007), because IFNα4 has stronger antiviral effects (Gerlach et al., 2009) and was among the IFNs with the most profound effect on CD8^+^ T-cells, with respect to anti-proliferative effects, improved cytokine production and cytotoxicity (Dickow et al., 2019). We decided to not include additional IFN subtypes which eventually have diverse biological functions (Zwarthoff et al., 1985; Hardy et al., 2004), which might be related to different binding affinities to the two IFNAR subunits (Jaks et al., 2007; Lavoie et al., 2011) as well as their ability to activate different downstream signaling pathways (Cull et al., 2003). The induction of distinct type I IFN-stimulated gene (ISG) expression patterns for each IFNα subtype (Li et al., 2017) contribute to their individual responses (Li and Sherry, 2010; Moll et al., 2011). Since one possible application of synthetic IFNARs are reinfused cytotoxic T-cells for viral but especially tumor defense, we were interested in the similarities of IFNα4 and our synthetic IFN system. Our transcriptomic analysis showed a high (99.91%) overlap of overall gene regulation including typical ISGs for viral defense. Moreover, we showed efficient VSV clearance in MC57 cells after stimulation of the synthetic IFNARs. Systemic application of type I IFNs in tumor therapy to enhance CD8 T-cell responses is, however, only resulting in short-term anti-tumor action because of negative effects during prolonged IFN signaling by inducing immunosuppressive factors such as PD-L1 and IL-10 (Zhou et al., 2020). Cell-type restricted type I IFN signaling using synthetic IFNs might prevent immunosuppression because these IFN actions might not be directly caused by action of type I IFNs on CD8 T-cells but by collateral cellular activation within the tumor environment.

Using our synthetic murine IFNARs, we re-evaluated the intracellular signaling with respect to STAT1/2 signaling. A previous study suggested that for murine IFNAR2, a single mutation in Y510 (the equivalent to human Y512) almost completely abolished STAT1/2 phosphorylation and biological activity, whereas a second tyrosine, Y335 (the equivalent to human Y337), plays a minor role (Zhao et al., 2008). Using deletion variants of our synthetic IFNARs, we showed that synthetic IFNAR1 did not contribute to STAT1/2 signaling directly. The main function of synthetic IFNAR1 was to facilitate Janus kinase activation, followed by synthetic IFNAR2 dependent STAT1/2 activation. Introduction of the single and double loss-of-function point mutations Y335F and Y510F in synthetic IFNAR2 and combination with full-length and deletion variants of synthetic IFNAR1 verified the major function of Y510 for STAT1/2 activation and devised a minor role to Y335. We also generated the synthetic human deletion variant IFNAR2Δ347 which was co-expressed with full-length synthetic human IFNAR1. In good agreement with the murine synthetic deletion equivalent IFNAR2Δ345, this combination of human receptors failed to induce STAT1/2 phosphorylation. In the synthetic deletion variant IFNAR2Δ347, the conserved tyrosine 512 and the non-conserved tyrosine 411 were deleted. Also the constitutive STAT2-binding site was deleted in synthetic IFNAR2Δ347, whereas Jak1 binding and activation was not affected (Shemesh et al., 2021). We decided to only test the synthetic hIFNAR2Δ347 deletion variant and not generate single tyrosine mutants for human IFNAR2 because it was previously shown that single mutations of each of the seven tyrosines including Y512 in human IFNAR2 did not result in any change of activity. Moreover, also simultaneous mutation of all seven tyrosines did not completely abolish STAT signaling (Shemesh et al., 2021), albeit this was not seen in another study (Wagner et al., 2002). We were more interested to confirm the important role of synthetic human IFNAR2 in STAT1/2 phosphorylation than to assign a certain tyrosine residue to STAT signaling. However, future work using our synthetic human IFNARs might contribute to unravel this controversial STAT activation pattern.

Using CC and GG synthetic ligands, we also tested the biological activity of homodimeric IFNAR1 and IFNAR2 complexes. IFNAR1 homodimeric complexes *via* EpoR_ECD_-IFNAR1_ICD_ fusion proteins were previously shown to activate Tyk2 but no STAT2 phosphorylation (Krishnan et al., 1996). Moreover, homodimeric EpoR_ECD_-IFNAR2_ICD_ complexes were biologically active, albeit with lower efficacy compared to the natural receptor complex (Pattyn et al., 1999). Therefore, we expected activation of signal transduction also by homodimeric IFNAR2 but not by IFNAR1. Since intracellular tyrosine motifs for STAT binding within IFNAR1 are missing, IFNAR1 cannot directly contribute to STAT signaling. Therefore, we did not observe any STAT1/2 phosphorylation following induction of homodimeric murine and human IFNAR1 complexes. Albeit rather unlikely (Urin et al., 2019), we cannot not exclude that other than STAT, p38, Akt or ERK signaling cascades might be triggered by homodimeric IFNAR1 complexes (Krishnan et al., 1996; de Weerd et al., 2013). We observed STAT1 and STAT2 phosphorylation following homodimerization of murine and human IFNAR2 in HEK293 cells which was as strong as induced by heterodimeric IFNAR2 complexes. For unknown reasons, homodimeric activation of murine and human IFNAR2 complexes was lower in murine Ba/F3-gp130 cells compared to heterodimeric IFNARs as shown by Western blotting and in inhibitory proliferation assays.

Synthetic redesign of murine and human IFNARs exemplified that synthetic IFN receptors might be of good use to unravel general IFNAR signaling because they phenocopied natural IFNAR signaling. In the case of murine IFNARs, synthetic murine IFNARs independently verified the importance of Y510 and Y335 for STAT1/2 activation in a background-free cellular environment. Here, signaling of endogenous and synthetic IFN signaling was analyzed and compared within the same cell lines, natural IFNARs were activated by type I IFNs and synthetic IFNARs by synthetic cytokine ligands. Other models rely on knock-downs or knock-outs of the natural cytokine receptors or on cells that normally do not respond to the respective cytokine at all, making site-by-site comparisons in one cell line impossible. Our synthetic system might also be useful to analyze the biologic consequences of patient-derived non-synonymous single nucleotide variants (SNVs) in IFNARs.

## Data availability statement

The datasets presented in this study can be found in online repositories. The names of the repository/repositories and accession number(s) can be found at: https://www.ncbi.nlm.nih.gov/geo/, GSE202839.

## Author contributions

NZ, NC, FV, JD, HX, and EZ: formal analysis, validation, and investigation. DF: supervision and data curation. BK, HA-H, SM, and PL: resources. JS: visualization and funding acquisition. JS and DF: conceptualization and project administration. NZ, JS, and DF: writing—review and editing. DF, HX, and BK: methodology. NZ and JS: writing—original draft. All authors contributed to the article and approved the submitted version.

## Funding

This work was funded by a grant from the Deutsche Forschungsgemeinschaft (RTG1949) and the Jürgen Manchot Foundation.

## Conflict of interest

The authors declare that the research was conducted in the absence of any commercial or financial relationships that could be construed as a potential conflict of interest.

## Publisher’s note

All claims expressed in this article are solely those of the authors and do not necessarily represent those of their affiliated organizations, or those of the publisher, the editors and the reviewers. Any product that may be evaluated in this article, or claim that may be made by its manufacturer, is not guaranteed or endorsed by the publisher.
